# Management of feeding Problem in a Patient with Cleft Lip/Palate

**DOI:** 10.5005/jp-journals-10005-1351

**Published:** 2016-06-15

**Authors:** Mridula Goswami, Babita Jangra, Urvashi Bhushan

**Affiliations:** 1 Professor and Head, Department of Pedodontics and Preventive Dentistry Maulana Azad Institute of Dental Sciences, New Delhi, India; 2 Postgraduate Student, Department of Pedodontics and Preventive Dentistry Maulana Azad Institute of Dental Sciences, New Delhi, India; 3 Postgraduate Student, Department of Pedodontics and Preventive Dentistry Maulana Azad Institute of Dental Sciences, New Delhi, India

**Keywords:** Cleft lip/palate, Feeding appliance, Pediatric dentistry.

## Abstract

In a child with cleft lip and/or palate, nutrition is the first priority as for any other child. These children have specific physical limitations. To fulfill their nutritional requirement, these children need modifications in order to thrive and grow. Failure to adjust to these needs could place the children into a potential life-threatening situation. One of the immediate problems to be addressed in a newborn with cleft lip/palate is difficulty in feeding. Nasal regurgitation and choking are common because of inability of the palate to separate the nasal and oral cavities. The case presented here discusses the management of feeding problem in the infant with cleft lip/palate.

**How to cite this article:** Goswami M, Jangra B, Bhushan U. Management of feeding Problem in a Patient with Cleft Lip/ Palate. Int J Clin Pediatr Dent 2016;9(2):143-145.

## INTRODUCTION

Clefts of the lip and palate are the most common congenital deformities involving the orofacial region. Incidence is 0.28 to 3.74 per 1,000 live births globally.^1^ In India, the incidence of cleft lip and palate ranges from 0.25 to 1.56 per 1,000 live births.^2^ Cleft lip/palate together accounts for approximately 50% of all cases, whereas isolated cleft lip and cleft palate each occur in about 25% of cases.^1^ The problems associated with individuals with a cleft lip/palate affect the functions performed by the oral and nasal cavities.^3^ The oronasal communication diminishes the ability to create negative pressure necessary for suckling.^4-7^ The feeding process is also complicated by nasal regurgitation of food,^5-7^ excessive air intake that requires frequent burping and choking.^7^ Feeding time is significantly longer and fatigues both baby and mother.^8^

The feeding plate obturates the cleft and restores the separation between oral and nasal cavities. It creates a rigid platform toward which the baby can press the nipple and extract the milk.^4^ It facilitates feeding,^3^ reduces nasal regurgitation,^5,6^ reduces the incidence of choking, and shortens the length of time required for feeding.^4-6^ The obturator also prevents the tongue from entering the defect^4-9^ and interfering with the spontaneous growth of palatal shelves toward the midline. Feeding plate restores the basic functions of mastication, deglutition, and speech production until the cleft lip and/or palate can be surgically corrected. The case presented here is the infant with cleft lip/palate to whom feeding appliance was fabricated to facilitate feeding.

## FEEDING APPLIANCE DESIGN

The feeding appliance consists of autopolymerizing acrylic resin base and two wire components made with 0.8 mm stainless steel. One end of these wires is embedded in autopolymerizing acrylic resin base and the other end with hooks is free and these ends are engaged in head cap of the patient.

## CASE REPORT

**Fig. 1:**
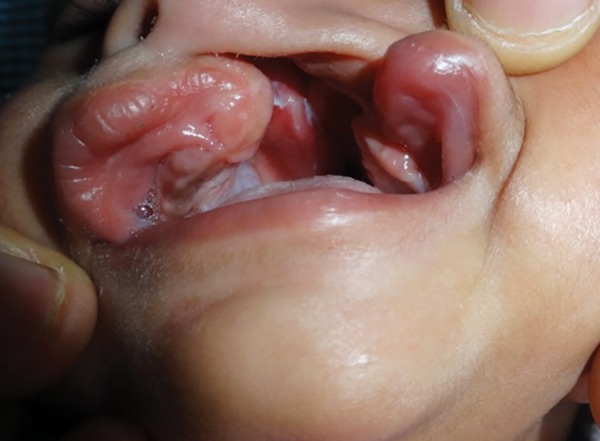
Intraoral defect in soft and hard palates involving alveolar process on left side of the premaxillary area

A 6-month-old infant was referred to the Department of Pedodontics and Preventive Dentistry, Maulana Azad Institute of Dental Sciences, New Delhi, with the chief complaint of difficulty in feeding mother’s milk and nasal regurgitation. The medical history of the child and parents was noncontributory. The family history was also noncontributory. On extraoral examination, there was defect in lips and alveolus. Intraoral examination of the child revealed a cleft involving soft and hard palates and the alveolar process on left side of the premaxillary area (Veau classification, Class III) (Fig. 1).

## PROCEDURE

**Fig. 2:**
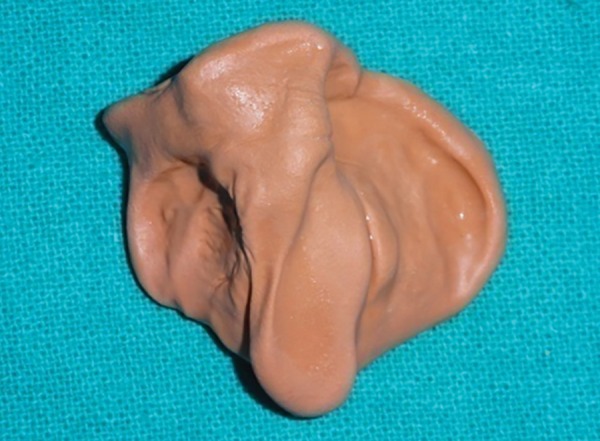
Preliminary impression

**Fig. 3:**
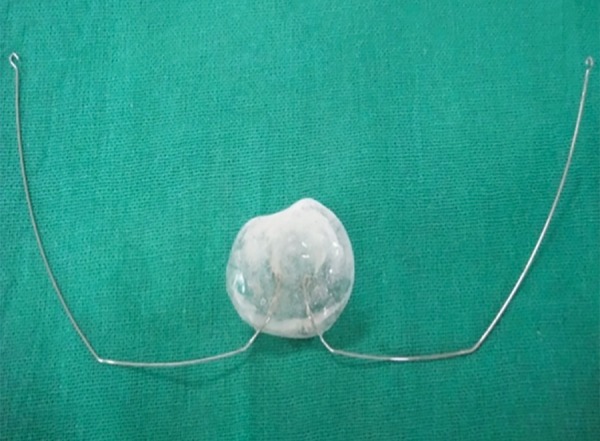
Feeding appliance

**Fig. 4:**
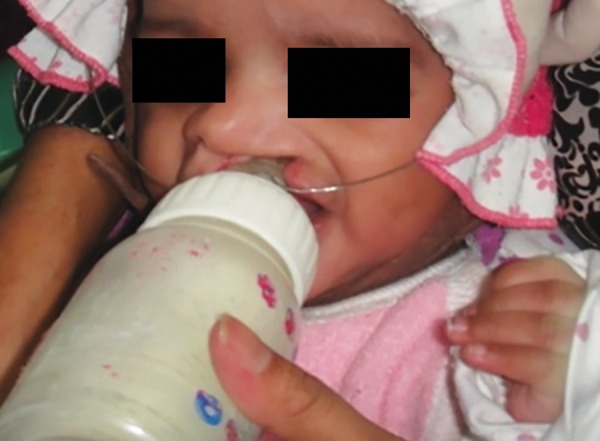
Feeding with appliance

A low-fusing compound was used for preliminary impression of the maxillary arch (Fig. 2). The infant was held with his face toward the floor in order to avoid aspiration. Also, it was noted that the infant was crying during the impression-making procedure. This thus ensured a patent airway continuously throughout the procedure. Custom tray was then fabricated by using autopolymerizing acrylic resin and secondary impression was made using alginate. It should be ensured that no residual material is left in the oral cavity. For obtaining good details, beading of impression was done. Final stone model was produced and all the undercuts were blocked. The feeding plate was fabricated on the dental stone model with autopolymerizing acrylic resins. Two 0.8 mm stainless steel wires were incorporated in the appliance for holding it in position during feeding (Fig. 3). These wires were engaged in the head cap of the patient, which had hooks with elastics. Finally, the appliance was placed in the oral cavity and the child was fed (Fig. 4). It needs to be ensured that the wire components do not interfere in lip movement while feeding.

## DISCUSSION

The infant born with a cleft has similar nutritional requirements as other infants born without a cleft, as long as no other systemic issues are involved.^10-12^ The main priority during the first few months of life for all infants is to ensure adequate nutrition.^13^ Feeding difficulties often experienced by infants with clefts that increase the problems with providing adequate nutritional intake include nasal regurgitation, poor suction, excessive air intake, frequent burping, and prolonged feeding times.^12^ The placement of feeding type of appliance is intended to facilitate the infant’s ability to create sufficient negative pressure that would allow adequate sucking and decrease the amount of fluid that flows back out through the nasal cavity rather than being swal-lowed.^14^ To overcome feeding problem, various feeding methods have been recommended and some others have advocated specific feeder for use in some or all clefting conditions.^15^ Many studies have reported enhanced feeding with plate in shorter feeding time.^15^ The major advantage of the feeding plate is to enhance the child’s ability to obtain nourishment during early stage.^1^ The main drawback of these feeding plates is the repeated requirement of fabrication of new ones because of growth and maintenance of good oral hygiene.^15^ Further comprehensive management of children born with cleft lip/palate is best accomplished by the multidisciplinary team approach, which not only benefits the patient, but provides a significant avenue for the understanding of diagnosis and management considerations. A pediatric dentist is involved frequently in the care of cleft lip/ palate children.^12^ They are involved at a much earlier age, even before the presence of dentition. Across all program types, it is evident that traditional services like preventive care and restorative dentistry are provided most frequently by pediatric dentists as a part of cleft palate Team.^11^ However, prompt intervention by fabrication of feeding plate can eliminate the immediate problems of proper nourishment and prevention of infections for the already debilitated infant. Thus the pedodontist is responsible for overall dental care of the patient. The infants born with a cleft may require modifications in feeding practices prior to surgical closure of the defect. Early referral for dental care should be encouraged in these children because they demonstrate higher dental needs. Parents should be educated regarding causes and methods to reduce dental disease that help to decrease its incidence and help these children require less invasive and difficult rehabilitation therapy.^14^
